# The interplay between bacterial biofilms, encrustation, and wall shear stress in ureteral stents: a review across scales

**DOI:** 10.3389/fruro.2023.1335414

**Published:** 2024-01-16

**Authors:** Pedro Amado, Shaokai Zheng, Dirk Lange, Dario Carugo, Sarah L. Waters, Dominik Obrist, Fiona Burkhard, Francesco Clavica

**Affiliations:** ^1^ARTORG Center for Biomedical Engineering Research, University of Bern, Bern, Switzerland; ^2^Department of Neurology, Inselspital, Bern University Hospital, Bern, Switzerland; ^3^The Stone Centre at Vancouver General Hospital, Department of Urologic Sciences, University of British Columbia, Vancouver, BC, Canada; ^4^Nuffield Department of Orthopaedics, Rheumatology and Musculoskeletal Sciences (NDORMS), University of Oxford, Oxford, United Kingdom; ^5^Mathematical Institute, Radcliffe Observatory Quarter, University of Oxford, Oxford, United Kingdom; ^6^Department of Urology, Inselspital, Bern University Hospital, University of Bern, Bern, Switzerland

**Keywords:** urology, ureteral stents, biofilm, bacteria, encrustation, microfluidics, wall shear stress, fluid mechanics

## Abstract

Ureteral stents are hollow tubes that are inserted into the ureter to maintain the flow of urine from the kidney to the bladder. However, the use of these indwelling stents is associated with potential complications. Biofilm, an organized consortium of bacterial species embedded within a self-producing extracellular matrix, can attach to the outer and inner surfaces of ureteral stents. Furthermore, encrustation - defined as the buildup of mineral deposits on the stent surface - can occur independently or in parallel with biofilm formation. Both phenomena can cause stent obstruction, which can lead to obstructive pyelonephritis and make stent removal difficult. Understanding the influence of flow on the development of biofilm and encrustation and the impact of small mechanical environmental changes (e.g., wall shear stress distribution) is key to improve the long-term performance of stents. Identifying the optimal stent properties to prevent early bacterial attachment and/or crystal deposition and their growth, would represent a breakthrough in reducing biofilm-/encrustation-associated complications. This review identifies the most prevalent bacterial strains and crystal types associated with ureteral stents, and the process of their association with the stent surface, which often depends on patient comorbidities, stent material, and indwelling time. Furthermore, we focus on the often-overlooked role of fluid dynamics on biofilm and encrustation development in ureteral stents, across a range of physical scales (i.e., from micro- to macro-scale) with the aim of providing a knowledge base to inform the development of safer and more effective ureteral stents.

## 1 Introduction

Ureteral stents are indwelling medical devices commonly used to maintain the patency of the obstructed ureter to preserve/restore urine flow from the kidney to the bladder (1). A typical ureteral stent is a thin-walled polymeric tube with a length of 20-30 cm and an outer diameter ranging from 1.60 mm to 2.67 mm (2). A stent is equipped with small circular openings, known as side holes, that are often positioned throughout its length, allowing exchange of flow. Despite their widespread use, more than 80% of patients experience stent-associated complications, negatively impacting their quality of life and the healthcare resources (3, 4). The most common complications associated with ureteral stenting are: (i) encrustation and biofilm formation, potentially leading to occlusion and urinary tract infections (UTIs) (5, 6); (ii) vesicoureteral reflux (VUR), which involves the retrograde flow of urine from the bladder into the ureter (7); and (iii) loss of ureteral peristalsis, irritation of the urothelial wall, and haematuria (8). This review centers on biofilm and encrustation as the primary causes of failure in long-term ureteral stenting (i.e., >six weeks). In short-term stenting the failure rate is low, and frequent urination, urinary urgency, and flank pain are the most common complications. Moreover, even if encrustation and biofilm can form within days, in some cases, forgotten stents with long placement time (e.g., 15 years) remain unobstructed (9). Biofilm is defined as a complex and organized consortium of surface-associated bacteria that are embedded within a self-produced matrix of extracellular polymeric substances (EPS) and grow in this protective matrix (10). We refer to encrustation as the build-up of mineral deposits/crystals, or other inorganic solid material, on the surface of ureteral stents or within the matrix of a biofilm.

Over the years, practice-driven stent design, material, and coating, as well as physics-driven design, have evolved to reduce the risk of complications (11). A double-J (DJ) stent design characterized by the presence of ‘pigtail’ coils at both extremities was introduced to prevent stent migration upon insertion (12). Recent clinical studies refer to pigtails as the most prone regions to developing encrustation in stented patients with urolithiasis (5, 13). In addition, other *in vitro* studies point to the side holes as the primary location of early-stage biofilm formation and crystal deposition due to the presence of laminar vortices and low wall shear stress (WSS) levels (14, 15).

Regarding the ureteral stent material, silicone appears to have superior long-term performance against encrustation and biofilms when compared to other alternative polymers (16). However, the lower tensile strength and higher surface friction associated with silicone can pose challenges during stent placement and removal, contributing to a preference for polyurethane stents (17). Coating the stent surface to decrease encrustation has also been explored; specifically, heparin (18), amorphous diamond-like carbon (19) and hydrogel coatings have shown promising performance (16). Antimicrobial agents, like triclosan and silver, have also shown positive outcomes against biofilm formation but have raised concerns over the potential development of antimicrobial resistance (11, 20).

In parallel to stent coatings and materials, fluid dynamics-driven design is an active research area that is being pursued with the aim of reducing encrustation and biofilm formation in stents. Some studies focused on fluid mechanical changes due to different stent sizes (2, 21–23), and the diameter (2, 14) and spatial distribution (24, 25) of side holes using mathematical modeling (26–28), computational fluid dynamics (CFD) (21–25, 29–31), and *in vitro* flow systems (2, 14, 22). However, only a limited number of studies have analyzed the interplay between flow metrics (such as WSS), crystal deposition (14, 32, 33) and bacterial attachment (34) within a stented ureter. Furthermore, translating computational and experimental findings into actual stent improvements is a challenging endeavor that is often ignored or underestimated.

This comprehensive review aims to introduce and discuss the methods used to identify, quantify, localize, and manage biofilm and encrustation in ureteral stents. It first describes the bacterial species involved in biofilm formation, the process of crystal formation, and the interplay between biofilm and encrustation. It subsequently discusses ways to prevent or minimize the growth of biofilms on ureteral stents that are specifically optimized based on fluid dynamics. These include methods based on computer simulations and experimental tests to spatially resolve relevant flow metrics, particularly WSS at different dimensional scales. Finally, it identifies a range of WSS values acting on the stent surface and elucidates the impact of WSS on biofilm formation and crystal deposition.

## 2 The dual threats: microbial colonization and encrustation

### 2.1 Biofilm formation

Like many other medical devices, ureteral stents can be a site for the adhesion and proliferation of bacteria and the deposition and nucleation of inorganic crystals. Upon insertion, ureteral stents make contact with urine, blood (in some cases) and uroepithelia, which creates a reversible accumulation of proteins, polysaccharides, and macromolecules on the stent surface. This process has been reported to take place within minutes after stent placement (35, 36). Initially, the Tamm-Horsfall glycoprotein, [one of the most abundant proteins in urine], polysaccharides, and other molecules, are absorbed by the surface of ureteral stents, forming a layer known as the conditioning film (37). Among the many proteins found in the conditioning film, Elwood and colleagues (38) reported that genitourinary cytokeratins (i.e., a group of proteins expressed in the cells of the genitourinary system) play a significant role in film formation. Haemoglobin and inflammatory proteins have also been identified on the surface of ureteral stents removed from patients (38).

In the second phase, irreversible deposition of proteins, extracellular polymeric substances, and bacteria takes place. Although one clinical study (38) suggested that the existence of a conditioning film does not lead to elevated levels of bacterial adhesion and colonization, others have shown that bacteria have a greater affinity for attaching to the different components of the conditioning film than to the stent material itself, suggesting that this film plays it plays a critical role in facilitating bacterial adhesion (39, 40). This finding underlies the necessity to explore new techniques to prevent the conditioning film from forming in the first place, in addition to preventing bacterial attachment to the stent material.

When attaching to abiotic surfaces, bacteria tend to adhere through non-specific interactions, such as covalent bonds, electrostatic forces, van der Walls forces and acid-base interactions (41). When bacteria attach to surfaces through electrostatic interactions, the binding is weak, and cells can detach when subjected to shear forces. To withstand shear forces and possible electrostatic repulsion in proximity to surfaces, bacteria have developed thin, hair-like, non-flagellar appendages made of fimbria adhesins that help them attach (42). For *Escherichia coli*, fimbriae allow an irreversible attachment of bacteria through the binding of fimbrial adhesin FimH to the mannose absorbed on the surface (43). These strong catch-bond interactions between bacteria and polysaccharides on the surface influence the initial stages of bacterial colonization and increase in number with exposure to urine flow (44). When subject to higher shear stress levels (0.1 – 13Pa), biofilms tend to grow in a dense, flat monolayer structure, while they develop a thicker multilayer structure for lower shear stresses (<0.01) (45, 46).

Furthermore, most micro-organisms can produce EPS consisting of polysaccharides, proteins, nucleic acids, lipids and other organic and inorganic substances (47), forming a protective ‘capsule’ surrounding the bacteria. The EPS plays a crucial role in maintaining the integrity of the biofilm, by retaining water, and exhibiting sorption properties, facilitating nutrient uptake (47). This matrix can also capture and dissolve substances from the surrounding environment, thereby providing nutrients to the biofilm cells. It also contributes toward biofilm adhesion to surfaces and facilitates cell-cell interactions, leading to colonization, growth and maturation (48). After reaching biofilm maturation, the EPS matrix functions as a barrier against environmental stimuli and antimicrobial compounds, posing significant challenges to biofilm eradication or removal (49). Jin and colleagues (50) used computational methods to demonstrate that biofilm formation is influenced by two main forces: the drag force that pushes the cells away from the surface by deforming the EPS matrix, and a tensile force that keeps the cells attached to the surface due to the fimbriae catch-bond interaction. However, environmental factors (temperature, pH, salinity and shear stress), surface properties (charge density, wettability, roughness, topography and stiffness), physicochemical properties of the cells (hydrophobicity, components and functional groups on the cell wall, proteins, nucleic acids, and extracellular polymeric substances), and microbial characteristics (bacterial strain, membrane charge, motility, and adhesion properties) contribute to and influence the process of biofilm formation and growth (36, 49).

While numerous factors contribute to biofilm development, bacteria remain the primary cause of its formation (a summary on biofilm formation in ureteral stents is provided in **Box 1**). Biofilms often comprise a combination of bacterial species, which compete and/or cooperate to form multi-species biofilms (51). To effectively combat biofilm formation in ureteral stents, it is crucial to identify and understand the specific bacterial species that thrive in this environment.

Box 1 **Summary of biofilm formation in ureteral stents.**

• Ureteral stents are susceptible to biofilm development.

• Conditioning film forms from proteins and polysaccharides on stent surfaces.

• Bacteria adhere to stents and the conditioning film.

• Bacteria use flagella for strong catch bonds to resist shear forces.

• EPS matrix provides protection and nutrients to bacteria.

• Biofilms are usually flat under high-shear stress and thick under low-shear stress.

### 2.2 The interplay between bacteria and crystals

In a clinical study, Waters et al. (26) reported that the presence of encrustation in ureteral stents does not directly correlate with the presence of bacteria. Indeed, encrustation can be found in the absence of bacterial species, as well as in the presence of either non-urease-producing species or urease-producing species, suggesting that focusing solely on antimicrobials as a preventative strategy against encrustation may not be sufficient (26). However, the type of crystals that adhere to the stent material may vary according to the presence or absence of bacteria (a summary on bacterial influence on crystals is provided in **Box 2**).

Box 2 **Influence of bacterial presence on crystal types in ureteral stents.**

• Crystal type on ureteral stents can vary based on the bacterial presence.

• In absence of bacteria, calcium oxalate monohydrate crystals are the main crystals in ureteral stents.

• In presence of bacteria, struvite and hydroxyapatite crystals predominate.

• Certain bacteria accelerate stent encrustation.

In the absence of bacteria, the main crystal constituent that appears on ureteral stents is calcium oxalate monohydrate (52, 53). High uric acid levels due to extreme ingestion of animal proteins can increase the formation of uric acid stones, decreasing the urine pH below 5, which contributes to the formation of calcium oxalate and carbonate crystals (54). Conversely, pregnancy increases the risk of calcium phosphate stones due to increased calcium excretion and elevated urine pH (55). In the presence of urease-producing bacteria, urea is catabolized, forming ammonia and significantly increasing the urine pH to alkaline conditions (52), inducing crystal formation due to reduced solubility of certain solutes. Once these crystals form, they can grow until they can no longer remain suspended in the urine. At this point, they precipitate on the ureteral stent surface (53) in the form of magnesium, ammonium, and phosphate, producing struvite (magnesium ammonium phosphate) and hydroxyapatite (calcium phosphate) crystals. Infections with *Proteus mirabilis* are particularly complicated, as this bacterium can catabolize urea six to ten times faster than other bacteria (56). However, even if it is often considered a problematic bacterium, a clinical study showed that its mere presence on ureteral stents does not translate to increased stent encrustation (26). Overall, these studies highlight the significance of the interplay between bacteria and crystal deposition in the development of encrustation. Bacteria-induced encrustation forms more rapidly and is often thicker. Since bacteria survive within the encrusting material, effective removal of encrusting fragments is key to preventing recurrence.

Furthermore, while some antimicrobial coatings can reduce biofilm formation on polymeric stents, thus enhancing their durability, the effectiveness of these coatings can be compromised by the conditioning film and subsequent crystal deposition (57). Since crystals have a higher affinity to various components of the conditioning film than to the stent material itself (39), it is crucial to address the problem of crystal deposition in parallel with biofilm reduction.

### 2.3 Analytical methods to identify bacteria and crystals

To detect the presence of bacterial biofilms and encrustation in stents and evaluate their composition and physical characteristics, adequate analytical methods are necessary (a summary of the main methods is provided in **Box 3**). Multiple methods exist for analyzing bacterial populations embedded within biofilms, including optical and fluorescence microscopy, scanning electron microscopy (SEM), 16S rRNA sequencing, quantitative polymerase chain reaction (qPCR), biochemical tests or other quantitative methods that identify the cells after biofilm disruption using sonication, vortexing, scraping, or other techniques. Despite comprehensive reviews of these techniques, the choice of method largely depends on the objectives of the research. At present, no agreed-upon standard method exists for accurately isolating and quantifying the bacterial populations within the biofilm (58).

Box 3 **Detection and analysis of bacterial colonization and encrustation in ureteral stents.**

• Sterile urine cultures may underestimate the bacterial colonization on stents.

• Stent culture after vortexing and sonication is more effective than standard urine culture.

• Techniques like 16S rRNA sequencing, qPCR, and SEM improve understanding of stent microbiomes.

• SEM with EDX is commonly used to identify crystal types in stents.

• SEM, weight measurement, OCT, and µCT are used to quantify stent encrustation.

In the presence of specific symptoms, urine cultures are the most commonly applied tool for diagnosing urinary tract infections (UTIs) and to guide patient management. However, several clinical studies have shown that a sterile urine culture does not rule out bacterial colonization of stents, defined as the process where microbial organisms adhere and accumulate on the surface of the stent (59–69). Lojanapiwat and colleagues (59) investigated the presence of bacteria on i) removed stents and ii) in urine samples from patients, respectively. They determined that the conventional urine culture (CUC) method only estimated bacterial growth on the surface of the stent in 69% of cases compared to the results obtained from the conventional stent culture (CSC) method. Ozgur et al. (61) found an even larger discrepancy, showing that CUC was only 10% accurate compared to CSC. This indicates that CUCs are less effective at detecting bacterial colonization on stents than CSCs. The stent microbiome possibly originates from the adhesion of microbes present in the urinary tract; however, over time, it develops into a distinct population (70). This indicates that the stent microbiota cannot be solely attributed to urinary bacteria, implying that urine composition cannot be used as the sole indicator of biofilm formation in stents (70). However, conflicting hypotheses exist in this regard. Even though the presence of bacteria on the stent does not always cause symptomatic urinary tract infections, there is a noticeable correlation between bacteria strains found in urine cultures and the ones that colonize the stent (71).

Mandakhalikar et al. (58) developed an effective way to remove biofilms from surfaces for subsequent analysis. Their method involved first the detachment of the biofilm by vortexing, then the exposure to low-frequency ultrasound waves (sonication), followed by an additional step of vortexing. Bonkat et al. (60) found that the identification of bacteria after the sonication process with a roll plate resulted in better accuracy than CUC alone, when used in patient-removed stents (64). A recent approach involving 16S rRNA sequencing, qPCR, scanning electron microscopy (SEM), and bacteria cultivation, has enhanced our understanding of microbiome diversity in ureteral stents (72); however, Corcoll et al. (73) showed that varying the DNA extraction method employed can vary the 16S rRNA sequencing results. Moreover, this last method does not distinguish between alive and dead bacteria since it is based on DNA isolation, while biofilms consist of both live and dead bacteria. It is essential to note that detecting microorganisms on indwelling ureteral stents heavily relies on the stage of the biofilm lifecycle and the analysis method used (74).

Existing methods for analyzing encrustation mainly focus on identifying crystal types present and their amount. SEM, followed by energy-dispersive X-ray spectroscopy (EDX), is one of the most commonly used methods to identify the type of crystals in ureteral stents. By combining these two techniques, researchers can determine the morphology of the crystal plaque while acquiring information about their chemical composition (75, 76). Other methods are used to determine the composition of encrustation, such as fluorescence microscopy, infrared spectroscopy combined with X-ray diffraction spectroscopy (77, 78), SEM followed by inductively coupled plasma mass spectrometry (ICP-MS) (79), and others. X-ray crystallography (80) can also reveal the molecular structure of crystals.

Besides identifying the crystal type, some studies also used SEM as a qualitative method to estimate the extent of encrustation on different sections of ureteral stents. An *in vitro* study performed by Cauda et al. (76) used this technique to compare different stent materials, qualitatively identifying those developing less encrustation.

Various methods to quantify encrustation on ureteral stents have also been reported. SEM (81) was used to manually segment the encrusted regions and calculate their surface area and volume. However, this method is time-consuming and has drawbacks since it requires cutting the stent to visualize its lumen, leading to potential bias and loss of crystal deposits. Another technique involves measuring the weight of the stent before and after dissolving organic and inorganic material with oxidative acid, which provides accurate quantification but lacks information about the spatial distribution of crystals in the stent (82). Optical Coherence Tomography (OCT) can detect encrustation on internal and external stent surfaces while quantifying the level of stent lumen occlusion (83). Recently, two studies used micro-computed tomography (μCT) with morphologic and semantic segmentation to quantify encrustation in stents retrieved from patients and obtain information about its spatial localization (5, 13). The application of μCT and deep learning in these studies simplified the visualization of the stent lumen, thanks to the time-efficient and automatic nature of the quantification process. However, all the described methods require the stents to be removed from patients, which introduces bias in the quantification of encrustation, particularly on the outer surface due to potential dislodging. To address this issue, discarding the encrustation on the outer surface from the analysis eliminated this bias but represents a limitation of this technique (5). In this context, combining a quantification method with a physiologically relevant *in vitro* flow model could serve as a testing platform to help identify regions of the stent prone to encrustation and enable testing of new stent materials and designs, as shown by Zheng et al. (84). A recent review covers the topic of experimental flow models in greater depth (85).

### 2.4 Key bacteria in biofilms: the significance of optimal removal time and encrustation

By employing the different techniques described in section 2.3, researchers could successfully identify and quantify specific bacterial species that are present in indwelling ureteral stents (a summary of the main bacterial species found in ureteral stents is provided in **Box 4**). **Table 1** explores these studies, selected from a systematic search on the Scopus database with specific keywords ((“Ureteral Stent” OR “Ureteric Stent” OR “Double-J”) AND (“Colonization” OR “Bacterial Biofilm”)). Studies that only used CUC were not reviewed, since CUC alone is deemed insufficient to resolve the stent microbiota. **Table 1** provides information on various aspects relating to the bacterial isolation and identification methods, stent indwelling time, the most prevalent bacteria identified, stent material, antibiotic therapy (if applicable), patient conditions, and the percentage of the analyzed ureteral stents associated with the presence of bacteria (colonization rate).

Box 4 **Common bacterial species, indwelling time effects, and research challenges in ureteral stent studies.**

• *Escherichia coli* and *Enterococcus faecalis*, are commonly found on ureteral stents.

• In the *Staphylococcus* group, *S. epidermis* is most prevalent, followed by *S. aureus*.

• *Streptococcus*, *Klebsiella pneumoniae*, *Proteus mirabilis*, and *Pseudomonas aeruginosa* are also frequently found on ureteral stents.

• Longer indwelling time increases microbial colonization of stents.

• Bacterial load and encrustation are normally low under six weeks.

• The high variability of the research methods used in the field, complicates the Interpretation of the results and the drawing of clear conclusions.

**Table 1:** Characteristics of ureteral stents and associated bacterial colonization.

Isolation and identification	Indwelling time	Primary organisms	Stent material	Antibiotics	Inclusive criterium	Colonization rate	Reference
**Stent culture; conventional methods**	27 - 73 days 1 - 48 days	*Enterococci*, *Proteus, Streptococci. Klebsiella, E. coli*, etc. *Enterococci, S. epidermis, E. coli, Candida, Streptococci*, etc…	Polyurethane	For some patients, 3-7 days after stent insertion	a, b, c, d, e	100% 69.3%	Riedl et al. (86)
**Stent culture; digital colony counter and conventional method**	7 - 120 days	*Escherichia coli*, *Streptococcus* spp.*, Pseudomonas* spp.*, Staphylococcus* spp.*, Klebsiella pneumoniae*, etc…	Polyurethane	Before insertion	b, c, f, g	47.2%	Shabeena et al. (87)
**Scrapping, mixing and sonication; conventional method**	2 - 36 weeks	*Enterococcus faecalis, Staphylococcus aureus, E. coli, Pseudomonas aeruginosa*	Polyurethane	Before and after insertion	b, f, g, h, i, j, k, l, m, n, o, p	28%	Keane et al. (88)
**Stent culture; conventional methods**	< 4 weeks 4 - 6 weeks 6 - 28 weeks Permanent	*E. coli, Enterobacter, Pseudomonas spp, Staphylococcus, Streptococcus, Proteus, Klebsiella*, etc.	———	Three days of oral antibiotic after stent insertion	g, p, q, r, s, t, u	33% 50% 54% 100%	Lojanapiwat et al. (59)
**Washing suspension (scrapping, mixing and sonication) and stent culture; conventional method**	0 - 13 days 21 - 27 days 56 - 83 days > 83 days	*Enterococcus species, E. coli, Neisseria subflava, Acinetobacter baumannii, Candida*, etc…	Percuflex	Short-term (2-3 days) antibiotic prophylaxis	b, e, f, v	0% 36% 43% 75%	Paick et al. (89)
**Washing suspension and vortexing; conventional method**	14 - 21 days 22 - 28 days 29 - 35 days	*Enterococcus spp, Klebsiella pneumonia. Staphylococcus, Candida spp, E. coli*	Polyurethane	No	m	25% 11% 44%	Sarier et al. (90)
**Washing suspension and vortexing; conventional method**	14 – 72 days	*Pseudomonas fluorescens, Staphylococci, Ralstonia pickettii, E. coli*	Silicone	A single dose before surgery	d, q, x	7.7%	Ozgur et al. (61)
**Washing suspension and vortexing; MALDI-TOF MS**	17 - 72 days	*Staphylococcus epidermis, E. coli, Enterococcus faecalis*	Polyurethane	No	a, e, k, z, aa	20%	Ulker et al. (91)
**Stent culture; conventional methods**	11 - 213 days	*Enterococcus species, Staphylococcus epidermis, Proteus mirabilis, Pseudomonas aeruginosa*, etc…	———	Before insertion	b, c, f, g, k, p, v	34%	Akay et al. (92)
**Sonication; SEM**	5 - 128 days	*E. coli, Epidermis, P. mirabilis, Enterococci*, etc…	———	For some patients, 6-20 days	b, c, ab	90%	Reid et al. (62)
**Stent culture; Siemens autoSCAN4**	1 - 12 weeks	*E. coli, Pseudomonas aeruginosa, Klebsiella pneumoniae, Enterococcus faecalis*, etc…	Polyurethane	0.5h before insertion and 48h after insertion	f, q, ac, ad	82.9%	Zhang et al. (37)
**Stent culture; conventional methods**	4 - 8 weeks	*Staphylococci, Enterococcus faecalis, Proteus mirabilis, Klebsiella pneumoniae, E. coli*, etc…	————	1 hour before pyeloplasty and removal	ae	63%	Neheman et al. (65)
**Stent culture; conventional methods**	15 - 60 days	*Pseudomonas aeruginosa, E. coli, Pantoea* spp.	————	Yes	a, b, ac,	60%	Gede Oka et al. (66)
**Washing suspension; conventional methods**	1 - 43 days	*Staphylococcus, E. coli, Candida, Streptococcus, Staphylococcus epidermis*, etc…	Polyurethane	Only before insertion	b, c, e, f, g, k	29.4%	Aydin el. al (71).
**Stent culture; conventional methods**	< 4 weeks 4 - 6 weeks > 6 weeks	*E. coli*	—————	5-12 days	—————	23.5% 33.3% 71.4%	Rahman et al. (67)
**Rolling plate; automated Vitek ID system and AP120E**	5 - 940 days	*E. coli, Staphylococcus* spp.*, Pseudomonas* spp.*, Enterococcus* spp.*, Candida* spp.	Silicone	No	b, r, s, af,	42%	Kehinde et al. (93)
**Stent culture; conventional methods**	10 - 540 days	*E. coli, Enterococcus faecalis, Klebsiella pneumonia, Staphylococcus hominis*, etc…	————	5 days before stent insertion	b, m, q	35.8%	Saouli et al. (94)
**Stent culture; conventional methods**	20 - > 90 days	*Staphylococcus aureus, Staphylococcus faecalis, Pseudomonas aeruginosa, E. coli, Staphylococcus epidermis*, etc…	Polyurethane	5 days after stent insertion	b, c, e, f, g	95.5%	Klis et al. (95)
**Stent culture; conventional methods**	8 - 150 days	*Pseudomonas, E. coli, Enterococcus, Staphylococcus aureus, Staphylococcus epidermis*, etc…	Polyurethane and c-flex	Only upon insertion	b, f, m, x	44.5%	Lifshitz et al. (69)
**Mechanical abrasion; 16S rRNA gene via qPCR**	3 - 6 weeks	reveal distinct urotypes, gram-positive bacteria	———–	Upon stent removal	b	70.6%	Buhmann et al. (72)
**Scrapping, mixing and sonication; conventional method**	< 30 days 31 - 90 days > 90 days	*Staphylococci spp, Enterococcus spp, Enterobacteriaceae, Candida*	Polyurethane	Upon stent insertion	c, d, m, q, others	27% 39% 54%	Bonkat et al. (60)
**Scrapping, mixing and sonication; conventional method**	2 - 60 weeks	*Enterococcus* spp. *Lactobacillus* spp. *E. coli, Candida* spp.	Polyurethane	30-60 min prior to removal	m	27%	Bonkat et al. (64)
**Stent culture; conventional methods**	14 - 110 days	*E. Coli, Staphylococcus Aureus, Staphylococcus saprophyticus, Staphylococcus epidermis, Pseudomonas* spp., etc…	Polyurethane	Prophylaxis at the time of insertion	Not specified	15.6%	Samir et al. (96)
**Pinhole method: stent culture and 16S rRNA gene via qPCR analysis**	2 - 6 weeks	*E. coli, Gardnerella vaginalis, Staphylococcus epidermis, Enterococcus* spp.*, Pseudomonas* spp., etc…	Percuflex	1 hour before stent insertion and secondary ureterorenoscopy	ag	12.9% - stent culture 18.1% - qPCR	Zumstein et al. (74)
**Stent culture; SEM, conventional methods**	30 - > 150 days	*Enterococcus faecalis, Staphylococcus epidermidis, Staphylococcus haemolyticus, Escherichia coli, Staphylococcus aureus*	Polyurethane	———	e	34.73%	Zeng et al. (97)
**Stent culture; conventional methods**	15 - 90 days	*E. coli, Klebsiella, Candida*	———	Before DJ-stent placement	a, c, q, ah	18%	Yeniyol et al. (98)
**Stent culture; conventional methods**	5 - 25 weeks	*Staphylococcus* spp.*, Enterococcus* spp.*, E. coli, Fungi, Klebsiella*, etc…	Polyurethane and silicone	Before DJ-stent placement, and in case of trauma, 3-5 days	b, c, e, n, aa, ai, aj	74.3%	Kozyrakis et al. (99)
**Stent culture; conventional methods**	14 - 120 days	*E. coli, Streptococcus* spp.*, Candida albicans, Proteus mirabilis, Serratia* spp.	———	5 days before stent insertion	a, e, f, n, p, q, ah, ai, aj, ak,	24.2%	Al-Ghazo et al. (100)

This table provides essential information regarding the isolation and identification of primary organisms on indwelling stents. It includes data on the ureteral stent indwelling time, the five main microorganisms identified, the stent material, antibiotic treatments administered (if applicable), the criteria for patient selection, instances of stent colonization rate, and the corresponding references. The described study criteria for patient selection (pathologies and procedures chosen for the studies) are as follows: a) Ureteroscopy stone extraction; b.) Urolithiasis; c.) Hydronephrosis; d.) Diagnostic ureteroscopy; e.) Malignant ureteral obstructions; f.) Ureteric stricture; g.) UPJ obstructions; h.) Papillary necrosis; i.) Lymphoma; j.) Myelofibrosis; k.) Pregnancy; l.) Re implanted ureter; m.) Renal transplant; n.) Retroperitoneal fibrosis; o.) Transitional cell carcinoma; p.) Ureteric injury; q.) Shock wave lithotripsy; r.) Post ureteroscopy and endarterectomy s.) Post open ureter lithotomy; t.) Post open pyeloplasty; u.) Anuria; v.) UVJ obstruction; x.) Endopylotomy; z.) Laser lithotripsy; aa.) Obstructive pyelonephritis; ab.) Renal failure; ac.) Percutaneous nephrolithotomy; ad.) Ureterovesical reimplantation; ae.) Minimally invasive pyeloplasty; af.) Prevention of steinstrasse; ag.) Secondary ureteroscopy; ah.) Open urolithiasis surgery. ai.) UPJ stenosis; aj.) Uretero-ureteral anastomosis: ak.) UVJ stenosis).

By analyzing these previous studies, it emerges that *Escherichia coli* is consistently identified as the predominant bacterial species found in ureteral stents retrieved from patients. *Enterococcus* species, particularly *Enterococcus faecalis*, are also identified in most of these studies. Among the *Staphylococcus* group, *Staphylococcus epidermis* is the most prevalent one, followed by *Staphylococcus aureus*. Other bacteria, such as *Streptococcus*, *Klebsiella pneumoniae*, *Proteus mirabilis*, and *Pseudomonas aeruginosa*, are also identified in multiple studies.

In addition to the predominant bacteria mentioned above, a few other species have been identified (although less frequently), such as *Enterobacter*, *Neisseria subflava*, *Acinetobacter baumannii*, *Ralstonia pickettii*, *Pantoea*, *Pseudomonas fluorescence*, *Staphylococcus hominis*, *Serratia species* and *Lactobacillus*. *Candida* were also found to be prevalent microorganisms colonizing ureteral stents. For simplicity, this review and **Table 1** only provide the five bacteria with the highest prevalence for each study. However, more than five bacterial species were found in most studies.

The positive correlation between indwelling time and microbial stent colonization has been extensively reported (60, 91). In a clinical study analyzing 93 stents from 71 patients, Riedl et al. (86) reported that all ureteral stents were colonized at 27 to 73 days from insertion, while 69% of stents were colonized at 1 to 48 days from insertion. Lojanapiwat et al. (59) reported a 33% colonization rate at <4 weeks and 100% at >28 weeks. Paick et al. (89) found no bacteriuria at <13 days and a colonization rate of 75% at >83 days. Other clinical studies have also shown consistent trends (60, 67, 90).

Despite the proven correlation between indwelling time and bacterial colonization, determining the optimal indwelling time for ureteral stent removal remains a subject of ongoing debate. Some clinical studies reported no bacteriuria within 2 weeks from stent insertion (59, 61, 87, 89) despite an *in vitro* study showing that bacteria can attach within 24 hours (62). By using the 16S rRNA method, it has been shown that the bacterial load is low (<10^4^ CFUs/ml) for short-term stenting of 3 to 6 weeks (72). While a low colonization rate at <6 weeks has been reported (61), in contrast, other studies have indicated significant colonization in the first 3 weeks (71). Thus, the identification of a ‘safe indwelling time’ is challenging, due to the independent nature of most studies, employing different detection methods, indwelling times, stent types, and variations in patients’ conditions and comorbidities.

Comorbidities such as diabetes mellitus and renal insufficiency have been identified as risk factors for bacterial colonization (66, 92). Pregnancy, female sex, diabetic nephropathy, and cancer have been reported as additional risk factors for stent failure (101), although there is conflicting evidence concerning sex (96). The use of antibiotics was reported to be ineffective on bacterial colonization rate in the long term, although it can reduce initial attachment (102). The microorganisms found on stents are multidrug-resistant to the most common antibiotics (63), with bacteria isolated from stents being more resistant than similar species before placement (93). This illustrates the issue with prophylactic antibiotic use with implanted stents and antibiotic administration at the time of stent placement. Stent type, material and coatings can also influence colonization rates, as described in several reviews (11, 103, 104). Due to the large parametric space involved, there is a lack of standardization across studies, which makes it difficult to draw definite and robust conclusions in this area of research (**Box 4**).

Consistently with bacterial colonization, longer indwelling time also increases the rates and frequency of encrustation (105). To the best of our knowledge, there is no consensus on the optimal length of placement for ureteral stents to minimize complications such as blockages due to encrustation. However, some clinal studies have suggested that the risk of morbidity related to encrustation is minimal when the stent is removed within 6 weeks (105, 106), despite encrustation occurring as early as 7 days, forming a crystal layer on both the inner and outer stent surfaces of the stent (75). Moreover, it was reported that luminal encrustation in stone patients occurred in 47.5% of cases with indwelling periods ranging from 6 to 12 weeks, while at >12 weeks, the incidence rate was 75%, significantly raising the risk of obstructions (106). Despite a high percentage of occlusions (due to encrustation) take place in the stent lumen up to 3 months (30%), only a small proportion of patients (4%) experienced clinically significant obstructions, i.e., blockage or impaired function of the stent that leads to clinical symptoms (106). This demonstrates that urine flow primarily occurs around the stent rather than through the lumen (2, 106). Nonetheless, even if the encrustations can occur just within a few weeks from stenting, retained and forgotten ureteral stents still bear the most extreme cases of encrustation (107).

Determining the ideal time for stent removal based on the existing literature has proven challenging due to inter-patient differences and comorbidities. Depending on the patient’s condition, a forgotten stent can become heavily encrusted, creating severe complications. However, a promising technique has emerged for real-time monitoring of encrustation using the quartz crystal microbalance technique, enabling the evaluation of encrustation’s surface area on the stent (108). Moreover, a recent stent prototype featuring a micro pressure sensor showed promising results in terms of its ability to detect obstructions in indwelling stents (109). In this direction, the integration of obstruction monitoring methods with quantification techniques holds the potential to determine the appropriate time for stent removal and potentially identify regions of the stent that are most problematic.

### 2.5 Distribution patterns of biofilm and encrustation in ureteral stents

Ureteral stents can exhibit different encrustation and biofilm patterns depending on the specific segment of the stent (a summary of the distribution patterns is provided in **Box 5**). A stent can be divided into its two pigtails (located in the renal pelvis and the bladder, respectively) and a straight central segment. This straight part can be subdivided into three regions: i) proximal section (near the ureteropelvic junction or UPJ), ii) middle section (within the middle ureter), and iii) distal section (near the ureterovesical junction or UVJ).

Box 5 **Location matters in ureteral stent bacterial colonization and encrustation.**

• Microbial colonization is more pronounced in the distal and proximal sections of ureteral stents.

• Encrustations are typically more visible in the proximal section, pigtails, and side holes.

• Colonization and encrustation patterns can vary based on the patient type.

• Future consideration should be given to developing patient-specific stents.

While one study (91) reported that bacterial colonization was present in all regions of patient-retrieved stents in 71% of cases, other reports have shown different results. For instance, Aydin et al. (71) showed that the colonization rate was the same for each part of the stent in 30% of the cases. Akay et al. (92) found bacterial colonization of the distal section in 34% of cases and of the proximal section in 29% of cases. Another clinical study (37) reported a colonization rate of 85% in the distal, 43% in the middle, and 67% in the proximal section. These findings demonstrate an increased prevalence of bacterial colonization in the distal section of the stent compared to the proximal one, which is also consistent with a study by Zhang et al. (37). Much like encrustation, bacterial counts are greater at the distal and proximal ends since these are areas in permanent contact with urine, unlike the middle parts where urine flow affects colonization (26). The observed difference in colonization rate between proximal and distal regions of the stent can thus possibly be attributed to the fact that the primary bacteria responsible for colonization originate from the bowel and perineum (26). Although the colonization rate tends to differ depending on stent location, microbial diversity does not differ statistically between proximal and distal sections (110).

Regarding encrustation, several quantification techniques have been used to identify the regions of the stent that are more subject to it. By measuring the weight of encrustation, in a clinical study, Sighinolfi et al. (82) showed that the kidney pigtail exhibited the highest level of encrustation, followed by the bladder pigtail. A qualitative analysis of stents retrieved from patients showed a higher number of encrusted stents (80), with a greater visual amount of encrustation (105), on the renal pigtail, followed closely by the bladder pigtail. Conversely, some reports suggested that both pigtails are comparably affected by encrustation (5, 75), while Rane et al. (111) found the bladder pigtail to be more encrusted than the renal pigtail. Yoshida et al. (13) instead reported that the straight part of the stent was the more encrusted region, which is in disagreement with most other studies in this area.

Bader et al. (83) discovered a significantly greater level of luminal occlusion in the proximal section of the straight part of stents removed from patients. Zheng et al. (5) showed that, in stone patients, the proximal section was significantly more encrusted than the distal section, while for kidney transplant patients no significant difference was found. Arkusz et al. (75) reported that the proximal and distal sections of the stent were the most susceptible to deposition of urea. These differing conclusions can be attributed to the absence of standard methods for quantifying encrustation on ureteral stents. Furthermore, even when the same techniques are used, patients with a different clinical history will likely lead to different encrustation patterns (5). The presence and type of comorbidities, in particular, are known to affect encrustation; for example, encrustation of the kidney pigtail is positively correlated with urolithiasis, whereas encrustation of the bladder pigtail has been correlated with urinary tract infections (82). Moreover, it has been reported that VUR could alter the pattern of encrustation (84), and that side holes are one of the regions most prone to deposition of encrusting material (5, 15). In this regard, the possibility of developing patient-specific stents should be considered in the future. To this end, future scientific efforts should focus on combining information on stent sections that are more prone to develop encrustation (also based on patient-specific conditions) with the analysis of local fluid dynamics in these regions.

## 3 Role of fluid mechanics in the formation of biofilms and encrustation at the micro- and macro-scale

### 3.1 Values of wall shear stress within the stented ureter

Fluid dynamics is known to significantly impact various biological processes such as bacterial initial attachment (112), biofilm formation (113, 114) and encrustation (32). WSS appears to play an important role in modulating these processes within ureteral stents (33, 34). However, despite several studies focused on simulating urine flow in the stented ureter, only a limited number (summarized in **Table 2**) have computed and reported the WSS values at different locations of a ureteral stent. These studies were identified using the Scopus database with specific keywords ((“Ureteral Stent” OR “Ureteric Stent” OR “Double-J”) AND “Shear Stress”). **Table 2** provides an overview of key parameters from these studies, including study design, stent dimensions, flow rate, and WSS ranges observed on both the internal and external walls of the stent and side holes.

**Table 2:** Summary of study parameters and wall shear stress value ranges in ureteral stents.

Study model	Inner diameter	Wall thickness	Side hole	Condition	Flow rate	Wall shear stress	Author
**CFD on stent on a chip. Model a stent section with a stent lumen, 2 side holes (SH) and a ureter.**	1.5mm	0.5mm	0.8mm	Extrinsic obstruction	1 mL/min	Internal wall – order of 10^-1^ Pa Active SH - 0.04 Pa Drops by 75% at the Passive SH Occluded cavity - 10^-4^ Pa	Mosayyebbi et al. (32) and De Grazia et al. (34)
					10ml/min	Active SH - 0.4 Pa Drops by >82% at the Passive SH Occluded cavity - ~0 Pa	
**CFD on stent on a chip. Model of a stent section with a stent lumen, 2 SH and a ureter.**	1.5mm	0.3mm	0.8mm and angled SH	Extrinsic obstruction	1 mL/min	Active SH (no angle) - 0.06 Pa Drops by 16% at angle 45° Drops by 12% at angle 90° Drops by 2% at angle 120° Passive SH (no angle) - 0.02 Pa Increases by 78% at angle 45° Increases by 58% at angle 90° Increases by 41% at angle 120°	Mosayyebbi et al. (14)
		0.5mm	0.8mm			Active SH (no angle) - 0.03 Pa Drops by 50% at the Passive SH	
		0.7mm	0.8mm			Active SH (no angle) - 0.012 Pa Drops by 17% at the Passive SH	
**Macroscale CFD on a 3D stent. Model of stent lumen, 42 SH and ureter.**	1.5mm	0.5mm	0.8mm	Unobstructed	1 mL/min	Proximal SH - 10^−5^ to 10^−4^ Pa Distal SH – around 10^−2^ Pa Internal wall - 10^−2^ to 10^−5^ Pa External wall - 10^−1^ to 10^−6^ Pa	Mosayyebbi et al. (33)
				Extrinsic obstruction		Proximal SH - 10^−5^ to 10^−3^ Pa Middle SH - 10^−3^ to 10^−1^ Pa Distal SH - 10^−2^ to 10^−1^ Pa Internal wall - 10^−1^ to 10^−5^ Pa External wall - 10^−2^ to 10^−6^ Pa	
**Macroscale CFD and Particle Image Velocimetry (PIV) on a 3D stent. Model of stent lumen, 8 SH holes and ureter.**	1mm	0.5mm	1.1mm	Unobstructed	1ml/min	Proximal SH - 10-4 to 10-3 Pa Middle SH - around 10-3 Pa	Zheng et al. (2)
			0.4mm			Proximal SH – around 10-3 Pa Middle SH - 10-4 to 10-3 Pa	
**2D Stent lumen, stent wall**	Vortek^®^ Tumor Stent (7F, Coloplast, Denmark)		Not modeled	Abrupt changes in the stent lumen	0.5ml/min	Stent internal wall < 0.070 Pa Cavity due to obstruction < 0.0007 Pa	Vogt et al. (31)

This table provides a summary of WSS values acting on ureteral stents, determined in previous research. The information reported includes the type of model used, inner stent diameter, wall thickness, presence of side holes (SH), specific conditions under which experiments were conducted, flow rates employed, WSS values, and the respective authors of the study. This succinct summary offers a quick reference to the essential parameters and details relevant to the flow analysis models discussed in the paper.

Firstly, microfluidic Stent-on-Chip (SOC) models (32) were employed to quantify the WSS distribution in a ureteral stent, and the effect of side hole shape and stent wall thickness on the WSS field. However, these values should be interpreted cautiously as the intrinsic simplifications of microfluidic models prevent them from reproducing the full complexity of the stented ureter (e.g., the stent and ureteral geometry, number of side holes, and reflux).

In an attempt to provide more physiological WSS values, Mosayyebi et al. (33) used CFD to compute WSS in a tapered stented ureter that mimicked the ureter of a pig, and identified variations in WSS levels depending on the longitudinal position along the ureter. They also evaluated WSS acting over the inner wall of side holes, as shown in **Table 2**. In this tapered model, the ureteropelvic junction (UPJ) had a larger diameter than the ureterovesical junction (UVJ). This configuration resulted in elevated WSS levels within the mid and distal sections.

A comprehensive CFD and particle image velocimetry study by Zheng et al. (2) employed a full-scale model to assess WSS distribution over the inner walls of side holes. In this study, the ureter was modeled as an unobstructed tube with a constant diameter. The mean WSS was higher in the first and last side holes of the straight part of the stent, while the side holes in the middle section exhibited lower WSS. These differences may be attributed to the passive nature of side holes in the middle section, since there is no significant flow exchange through these holes (‘passive’ side holes), whereas the first and last side holes promote such flow exchange (‘active’ side holes). Their findings also indicated that smaller side holes experienced significantly greater WSS levels, providing a potential means to mitigate encrustation and biofilm formation in these regions of the stent, due to the correlation between these phenomena and WSS.

Vogt et al. (31) conducted a small-scale CFD simulations on DJ ureteral stents, emphasizing the importance of utilizing smooth stent designs, since grooves and imperfections on the stent surface lead to stagnation zones (with low WSS levels) which can act as traps for crystals and promote encrustation.

By summarizing all the studies that reported WSS values at different locations of the ureteral stent in **Table 2**, our intention is to provide valuable insights into the fluid dynamics of the stent. The findings reveal that the proximal side holes exhibit WSS values below 0.001 Pa (2, 33), indicating relatively low to moderate fluid shear forces in this region. The WSS increases toward the middle side holes, with values ranging from the order of 0.0001 Pa up to 0.1 Pa (2, 33), suggesting a notable enhancement in fluid shear stress. The distal side holes also demonstrate higher WSS values, ranging from the order of 0.01 Pa up to 0.1 Pa (33). Concerning the stent internal wall, the WSS was reported to range between 10^-6^ Pa to 0.19 Pa (31), corresponding to a wide range of fluid shear forces.

The stent external wall experienced WSS values up to 0.11 Pa (33), which are lower than those acting on the inner stent surface. Cavities created by ureteric obstructions or stent defects can lead to stagnant regions where the WSS ranges from 0 Pa up to 0.00016 Pa (31, 32, 34). Moreover, the total range of WSS values for the entire stent is found to be from 0 Pa to 0.19 Pa, encompassing the whole spectrum of fluid forces experienced by the stent (a summary of the wall shear stress values in different locations of ureteral stents is provided in **Box 6**).

Box 6 **Wall shear stress values across various ureteral stent locations.**

• Stent design affects shear stress and encrustation patterns.

• Computational studies showed:

 o Proximal side holes show very low wall shear stress (WSS) below 0.001 Pa.
 o WSS increases toward the middle, ranging from 0.0001 to 0.1 Pa.
 o Distal side holes have higher WSS, between 0.01 and 0.1 Pa.
 o WSS varies from 10^-6^ Pa to 0.19 Pa on the stent’s internal wall.

In summary, accurate WSS measurements in stented ureters depend on various factors and the study design. Notably, none of the studies reviewed have considered the impact of reflux, peristalsis, and patient-specific comorbidities, which substantially influence WSS values and add complexities to a model. To better understand the true impact of WSS on ureteral stents, future research must diligently consider and incorporate these factors into their models (whether *in vitro* and/or *in silico*).

### 3.2 Impact of wall shear stress on ureteral stents’ blockage

At the micro-scale, the relationship between WSS and bacterial interactions with surfaces has been extensively explored in various domains (115) (see Section 4). However, this critical association remains largely unexplored in the context of ureteral stents. De Grazia et al. (34) conducted experimental investigations using a microfluidic SOC approach. Their results demonstrated that cavities, or stagnant regions with low WSS (i.e., below 0.04 Pa), were the primary sites of bacterial attachment, followed by side holes and the intra-luminal surface. This study marked the first attempt to examine bacterial attachment on ureteral stent architectures, quantitatively. The study revealed a correlation between bacteria coverage area, WSS, and the number of stagnant regions. However, *Pseudomonas fluorescens* was used as a bacterial model, which is not a highly prevalent bacterium in the urinary tract and may limit the clinical relevance of the study and its generalization.

Mosayyebi et al. (32) conducted a similar SOC study to investigate the impact of WSS on the deposition of encrusting particles. Their research revealed that the side hole close to the obstruction (active side hole) had a greater average WSS (0.04 Pa) and a greater exchange of flow, while the more distal one (passive side hole) had a lower WSS (0.01 Pa) with nearly stagnant flow. Notably, particle deposition was inversely correlated with WSS, i.e., areas with lower WSS were more prone to particle accumulation. Moreover, the same group (14) demonstrated that thinner stent walls and angled (i.e., streamlined) side holes could reduce particle deposits in stents as a result of increased WSS levels, particularly in the passive side holes.

Besides the SOC study, Mosayyebi et al. (33) also investigated the relationship between WSS and crystal deposition at the macroscale level, i.e. in a full-scale model of a stented ureter, using CFD and experimental tests. Their study stands out as the only macroscale model investigating this relationship, experimentally. It was observed that the accumulation of particles was more pronounced in the side holes situated in the proximal region of the stent compared to those in the distal region, as expected by the higher shear stress levels in the former.

To investigate encrustation in ureteral stents and its correlation with VUR, Zheng et al. (84) developed a macroscale *in vitro* model of the urinary tract with a programmable bladder to mimic the detrusor pressure waveforms during the bladder voiding stage. This was the first physical model of the urinary tract able to mimic reflux from the bladder to the kidney. Notably, this experimental configuration produced encrustation distribution patterns resembling those observed in stents retrieved from stone patients (5), supporting the hypothesis that the distal stent region is ‘cleaner’ because of a wash out effect caused by VUR. Although this study does not establish a direct correlation between VUR and WSS, this hypothesis about the role of reflux can be inferred from the work of Kim et al. (116): despite using non-physiological flow rates for urine flow, their full-scale CFD study showed that during the voiding stage, the average WSS at side holes located in the distal section is larger than in the middle and proximal sections.

Moreover, using computational and experimental modeling, Clavica and colleagues (15, 117) studied the flow field in the region located between an obstruction of the ureter lumen and the first side hole post-obstruction, using an artificial model of the obstructed and stented ureter. They observed the presence of low-velocity laminar vortices in the extra-luminal region between the obstruction and the side hole. They suggested that these vortices could lead to particle trapping and act as ‘seeding’ sites for encrustation. In this context, vortical flow has been shown to play a critical role not only in particle deposition but also in bacterial agglomeration, inducing biofilm formation and biofilm streamers, defined as filamentous structures that can extend into the surrounding environment (118).

**Figure 1:**
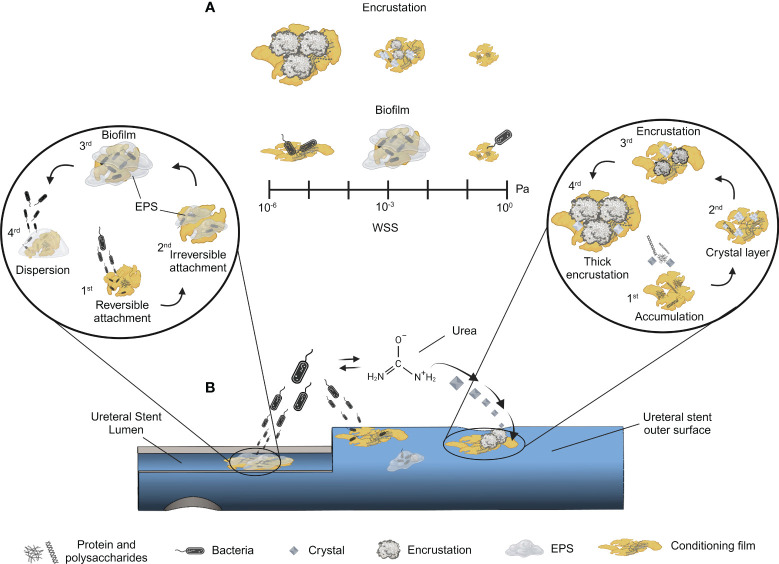
Sequential process of encrustation and biofilm formation in ureteral stents. Multi-stage development of biofilms and encrustation on ureteral stents are shown, highlighting the dynamic interaction with WSS. *Panel* **(A)** - A scale of WSS is included to show its impact on these phenomena: low WSS favours thicker encrustation, while higher WSS reduces it. Low shear conditions limit nutrient availability resulting in lower biofilm size, whereas medium shear provides optimal conditions for biofilm growth. Further increases in WSS lead to biofilm detachment. *Panel* **(B)** *-* Schematic of a colonized ureteral stent: the two insets represent the different stages of biofilm formation (left) and encrustation (right), respectively. Biofilm and encrustation can occur on both surfaces (stent lumen and stent outer surface). The stages for biofilm formation are: 1) reversible attachment through weak interactions, 2) irreversible attachment involving fimbriae, catch bonds, and EPS followed by 3) biofilm maturation, and 4) dispersion. The encrustation process is depicted starting from 1) protein and polysaccharide accumulation aiding conditioning film formation, 2) progressing crystal layer deposition, leading to 3) encrustation and 4) culminating in thick encrustation. In the middle of panel A, bacterial activity in the presence of urea is depicted, emphasizing its role in catabolizing urea into ammonia, which raises pH and crystal formation, enhancing encrustation. *Illustrations ere created with BioRender.com*.

To date, researchers have conducted microfluidic-based studies that correlate WSS with bacterial attachment or crystal deposition, treating each phenomenon independently. Macroscale analyzes that linked WSS only to crystal deposition have also been carried out. However, to the best of the authors’ knowledge, a comprehensive investigation relating flow dynamic analysis with biofilm and encrustation is still lacking in the literature – at either the microfluidic or macroscale level (a summary of the influence of wall shear stress in bacterial colonization and encrustation is provided in **Box 7**). **Figure 1** illustrates the multi-stage development of biofilms and encrustation on ureteral stents, highlighting the dynamic interaction between bacteria and encrustation influenced by WSS. To shed light on this, *in vitro* models, which have already been developed and established in the literature (85), could be perfused with both artificial urine and urinary bacteria.

Box 7 **Significance of wall shear stress in bacterial attachment and encrustation in ureteral stents.**

• Experimental studies showed that areas with low WSS (below 0.04 Pa) are primary sites for bacterial attachment, especially around passive side holes.

• Experimental studies showed that particle deposition is more common in areas with lower WSS.

• Stent design (e.g., thinner walls and streamlined side holes) influences shear stress and encrustation patterns, as shown experimentally.

• Particle accumulation is higher in proximal side holes (WSS < 0.001 Pa) compared to distal ones (WSS > 0.01 Pa), as shown experimentally.

• Experimental studies showed that vesicoureteral reflux (VUR) reduces encrustation.

• There is a lack of comprehensive studies integrating flow dynamics with biofilm formation and encrustation.

## 4 What can we learn from other research areas?

Formation of biofilm and encrustation represent significant challenges for ureteral stents, affecting patient’s health and therapeutic outcomes. Although studies directly correlating bacterial attachment and WSS in the urinary tract are only a few and very recent (34), valuable information can be gained from studies already conducted in other research areas, i.e., not directly linked to endourology. This section explores how findings in other fields could also be translated and extrapolated in the context of the distribution and location of bacteria on ureteral stents (a summary of the main findings is provided in **Box 8**).

Box 8 **Valuable insights into bacterial attachment and wall shear stress from related research.**

• Despite limited experimental studies directly linking bacterial attachment and WSS in the urinary tract, valuable insights are derived from other research areas.

• Microfluidics is used to explore how shear stress affects biofilm formation.

• Experimental studies showed that bacterial colonization decreases with WSS > 0.02 Pa, increasing detachment at higher shear.

• Different bacteria can exhibit different attachment rates depending on the level of WSS, as shown experimentally.

• Based on the information above, the development of fluid-mechanical-based stent designs, maximizing WSS to reduce bacterial attachment, should be encouraged.

In this section, we focus on the bacterial strains commonly associated with ureteral stents, namely *E. coli*, *Staphylococcus aureus* and *epidermis*, *Enterococcus faecalis*, *Pseudomonas aeruginosa*, *Klebsiella pneumoniae*, *Proteus mirabilis* and *Streptococcus*. These bacterial strains were selected based on the findings reported in **Table 1**. We report findings related to bacterial behavior within specific WSS ranges (0 Pa to 0.19 Pa), consistently with values reported in **Table 2**.

**Table 3** provides an overview of microfluidic studies that have established a correlation between WSS levels (within the range of values observed in ureteral stents) and the behavior of bacteria that are prevalent in the urinary tract. The studies were identified and selected using the Scopus database with specific keywords (“*” AND (“shear stress” OR “biofilm formation” OR “biofilm growth”) AND (“microfluidics” OR “microfluidic” OR “micro-fluidic” OR “flow chamber”), where * represent all the bacteria selected above).

**Table 3:** The impact of wall shear stress on biofilm formation in microfluidic experiments.

System Type	Organisms	Surface Materials	Stage of Biofilm	Range of Average WSS (Pa)	Channel dimensions	Main findings	Reference	
**Microfluidics (Parallel flow chamber)**	*Yeast cells*	Type 3 fimbriae removed from *E. coli* and *Klebsiella pneumoniae*	Adhesion	[0. 01-1.4]	L - 2.5cm; W - 0.25cm; H - 250μm	At the minimum shear stress level of 0.01 Pa, the accumulation of *yeast cells* on the surface was 5%, which increased to 60% when the shear stress reached 0.097 Pa. Further increase of shear stress lowers the accumulation.		Stahlhut et al. (119)
**Microfluidics (Micropillars)**	*E. coli*	PDMS	Adhesion	[0.065-0.259]	D – 15µm - 25μm; S -50 or 100μm, H- 20μm	Under fluid shear stress, the bacteria aligned parallel to the direction of the flow. Micropillars with larger diameter led to greater bacteria accumulation, regardless of the flow conditions.		Jin Hong Mok et al. (120)
**Microfluidics (Parallel flow chamber)**	*E. coli*	T-24 epithelial cells	Adhesion and detachment	[0.01– 1.15] [0.06- 18.46]	L – 1cm; W -2.5mm; H -250μm	The binding of *E. coli* to bladder T24 transitional cells and type IV collagen reached its highest level under minimal shear stress conditions and decreased with any increase in flow velocity. Once *E. coli* bound to host cells or collagen, they remained attached even when subjected to elevated shear stress.		Piatek et al. (115)
**Microfluidics (Parallel flow chamber)**	*E. coli*	PDMS and glass	Adhesion and proliferation	[0.00042 to 0.0315]	L – 30mm; W – 1mm; H - 250-1000μm	Biofilm formation on the surface was not observed when the shear stress exceeded a threshold value of 0.011 ± 0.002 Pa. Biofilms were prone to form in regions with low shear stress and spread from these locations toward areas with higher shear stress.		Thomen et al. (121)
**Microfluidics (Parallel flow chamber)**	*E. coli*	Glass, polymer brushes	Adhesion	[0.005 and 0.056]	————	The shear stress did not significantly affect the performance of the polymer brushes against bacterial adhesion.		Lopez-Mila et al. (122)
**Microfluidics (Parallel flow chamber)**	*E. coli*	PDMS	Adhesion and maturation	[0.0022 and 0.02]	L – 3cm; W – 1cm; H – 45μm	Unlike at 0.0022Pa, where the bacteria formed 3D rounded structures, at 0.02Pa the bacteria remained attached to the surface, but none of the tested strains formed mature biofilms.		Lim et al. (113)
**Microfluidics (Parallel flow chamber)**	*E. coli*, *Staphylococcus epidermidis*	PDMS with peptide-based coating	Adhesion	[0.01 and 0.024]	————	Introducing peptides to the smooth surface reduced *E. coli* adhesion by 58% at a shear stress of 0.01Pa and 43% at 0.024Pa.		Dolid et al. (123)
**Microfluidics (Parallel flow chamber)**	*E. coli*	PDMS and glass	Adhesion	[0.005 and 0.07]	L – 25.42cm; W – 1.6cm; H – 0.8cm	At shear stress of 0.005 Pa, the adhesion of cells on PDMS was found to be, on average, 1.7 times greater than on glass, with statistical significance (P < 0.05). At shear stress higher than 0.04Pa, a lower number of cells adhered to both surfaces.		Moreira et al. (124)
**Parallel flow chamber**	*P. aeruginosa, E. coli, C. tropicalis*	Glass and PEO-brush	Adhesion	[0.0000722 - 1.1]	L – 175mm; W – 17mm, H – 0.75mm	The initial deposition rates of *P. aeruginosa* and *E. coli* on both glass and the PEO-brush surface decreased as the shear rate increased. For *C. tropicalis*, the initial deposition rates remained relatively constant up to a shear stress of 0.07Pa, after which they started to decrease.		Roosjen et al. (125)
**Microfluidics (Parallel flow chamber)**	*Staphylococcus epidermidis*	PDMS	Adhesion and detachment	[0.04 - 0.15]	L – 22mm; W – 100μm; H – 100μm	High WSS likely restricted biofilm to a single layer. Dispersin B cleared most of it from the microchannel, leaving only some bacteria in low WSS corners.		Hyun Lee et al. (126)
**Microfluidics (Parallel flow chamber)**	*Staphylococcus epidermidis, Staphylococcus aureus, Pseudomonas aeruginosa*	Polyethylene oxide (PEO) coating and silicone rubber	Adhesion and detachment	[0.01 – 4]	L – 175mm; W – 17mm; H – 0.75mm	Biofilm uniformly coated the unaltered silicone rubber, while distinct clusters appeared on the brush-coated surface. Higher WSS enhanced biofilm detachment.		Nejadnik et al. (127)
**Microfluidics (Parallel flow chamber)**	*Staphylococcus aureus*, *Candida albicans*	PDMS	Adhesion	[0.025 - 0.074]	W – 180μm; H – 100μm	*S. aureus* cells proliferated more than *C. albicans* at 12 hours. Biofilm coverage decreased as WSS increased.		Tran et al. (128)
**Flow chamber**	*Pseudomonas aeruginosa*	Silicone rubber	Detachment	[0.005 - 0.025]	L – 35mm; W – 35mm; H – 0.6mm	Significant biofilm removal happened at local shear stress greater than ~0.018 Pa, and nearly all cells were removed from the flow cell surface at >0.019 Pa.		Zhang et al. (129)
**Microfluidics (Parallel flow chamber)**	*Pseudomonas aeruginosa*	PDMS	Adhesion and detachment	[0.0068 - 0.0852]	W – 300μm; H – 40μm	Biofilm formation peaked at a shear stress of 0.0170 Pa, with reduced formation at levels below or above this optimal WSS.		Park et al. (114)
**Microfluidics (Parallel flow chamber)**	*Pseudomonas aeruginosa*	Silicone-coated glass slides	Adhesion	[0.001 - 0.026]	L – 50mm; W– 10mm; H – 10 mm.	Initial cell attachment increased with low shear stress (up to 0.0035–0.005 Pa), yet higher shear stress reduced attachment.		Raya et al. (112)
**Microfluidics (Parallel flow chamber)**	*Pseudomonas aeruginosa*	PDMS	Adhesion and proliferation	[0.002 - 0.014]	L – 8000μm; W – 600μm; H – 250μm	Above a certain flow velocity, there was no occurrence of new biofilm formation and existing biofilm thickness decreased to nothing. A mathematical model was developed and validated for the highest flow rates imposed.		Janakiraman et al. (130)
**Microfluidics (Parallel flow chamber)**	*E. coli*	Glass	Adhesion and detachment	[0.000034 - 0.0034]	L – 3cm; W – 1cm; H – 0.0762cm	Shear stress caused significant detachment of nonmotile cells, intensifying with faster flow rates, while motile bacteria adherence grew with increasing fluid velocity.		McClaine et al. (131)
**Microfluidics (Parallel flow chamber)**	*Pseudomonas aeruginosa*	PDMS	Adhesion and detachment	[0 -0.1]	Several microstructures (circle, square, hexagon and triangle)	Microstructures improved diffusion and higher flow induced WSS, contributing to thinner biofilms.		Roh et al. (132)
**Microfluidics (Parallel flow chamber)**	*E. coli*	Glass, Peptide coated glass, Poly(L-lactic)	Adhesion	[0.007 – 0.08] (assuming viscosity of water)	————	At 15/s shear rate, the coating decreased cell adhesion by over 50%. Peptide-coated glass increased adhesion up to 30/s, decreasing it at greater shear rates.		Alves et al. (133)

This table provides information regarding the study’s parameters, including system type, organisms involved, surface materials (where PDMS denotes polydimethylsiloxane), stage of biofilm development defined as the adhesion phase (initial attachment), maturation (community of microorganisms and extracellular matrix) and detachment (removal of the biofilm), the range of average WSS levels measured in Pascal, and the dimensions of the microfluidic channels (L for length, W for width, and H for height). Additionally, it provides a summary of the main findings and references for further details.

These studies highlight the potential of microfluidics as a technology platform and modeling tool to investigate the complex interplay between shear stress and biofilm formation. Moreira et al. (134) found that bacterial attachment remained consistent between macro and microfluidic experiments, even with an 80-fold scale difference, while maintaining the WSS constant. This implies that microfluidic findings can be applied to a macroscale scenario under equivalent wall shear stress levels. Other flow conditions like Reynolds number or flow velocity could also be potentially investigated for their role in biofilm formation and growth. A review of different platforms to study biofilm formation under different flow conditions and WSS ranges, beyond the scope of the current paper, is provided elsewhere (135).

Stahlhut et al. (119) showed that fimbriae type 3, or MrkD, are specialized protein structures found on the surfaces of bacteria such as *E. coli* and *Klebsiella pneumoniae*. These structures play a pivotal role in yeast cell adhesion, with the most robust adhesion observed with the prevalent MrkD protein sequence C3091, showing enhanced binding within a WSS range of 0.01 to 0.097 Pa. Notably, other fimbriae variants exhibit varying attachment strengths; however, they follow a consistent trend of enhanced binding within a similar shear stress range, followed by diminishing adhesion beyond this threshold. Similarly, Raya et al. (112) explored the attachment of *Pseudomonas aeruginosa* to silicone-coated glass, revealing an increasing-decreasing trend of bacterial attachment with the increase of WSS, albeit with detachment starting at 0.005 Pa. Piatek et al. (115) reported that the binding of *E. coli* to bladder T24 transitional cells and type IV collagen reaches its highest level under minimal shear stress (0.01 Pa) conditions, and decreases with any increase in WSS. Notably, even when subjected to higher shear stress (0.59 Pa), some *E. coli* cells remain attached after binding to host cells or collagen, though detachment rates escalate with the magnitude of shear stress.

Park et al. (114) reported that *Pseudomonas aeruginosa* biofilm formation on PDMS was retarded at both WSS>0.017 Pa (due to shear) and WSS<0.017 Pa (due to lack of nutrients and oxygen). A WSS value of 0.017 Pa was reported to be the optimal shear stress level promoting the formation of a structured EPS while also providing a shield to the bacteria. Moreover, Zhang et al. (129) reported substantial biofilm detachment for the same bacterial species on silicone rubber surfaces at shear stress exceeding 0.018 Pa. Confirming these findings with *E. coli*, Lim et al. (113) reported that at 0.0022 Pa, bacteria form three-dimensional rounded biofilm structures. In contrast, at 0.02 Pa, the bacteria adhere to the surface but fail to develop mature biofilms. Furthermore, Janakiraman et al. (130) showed that biofilm thickness is significantly reduced with an increased flow rate.

The studies summarized in **Table 3** do not use materials that are commonly employed to manufacture ureteral stents. Different surface properties can result in varying degrees of bacterial adhesion, even upon exposure to the same WSS levels, as demonstrated by Moreira et al. (124). Their findings reveal that at a WSS of 0.005 Pa, bacterial adhesion on PDMS surpassed that on glass by 1.7 times. Conversely, at 0.04 Pa, most bacteria detached from both surfaces. Further research highlights the significant role that coatings and WSS play in the process of bacterial adherence, demonstrating that variations in surface properties lead to varied bacterial adhesion outcomes (123, 126, 127, 133, 134).

Validating these findings using materials that are typically used for ureteral stents (e.g. polyurethane, silicone, or specific surface coatings) could yield a more accurate determination of a WSS threshold that would prevent bacterial adhesion on indwelling urological devices. In this regard, some studies used polyurethane or associated coatings to verify the interplay between bacterial adhesion and WSS. However, only limited work has been conducted using WSS levels that are relevant to ureteral stents (136–140). As outlined in the previous section, some recent studies have highlighted a tendency toward bacterial colonization that is enhanced in the distal and proximal sections of stents (37, 92, 141). However, reflux and narrow regions (e.g., UVJ) in the distal segment of the ureter increase the WSS acting on the stent in this region (24). This increase in shear stress possibly contributes to the reduction of encrustation levels in this region compared to the proximal ones, as shown previously (84). Notably, the reported WSS in the distal section of the ureteral stent spans from 10^-2^ to 10^-1^ Pa. In comparison, those in the proximal section range from 10^-5^ to 10^-4^ Pa. Considering several reports suggesting the complete suppression or substantial reduction of biofilm thickness beyond 0.02 Pa, this becomes a possible reason for the observed phenomenon.

Furthermore, under identical flow conditions, different bacterial species exhibit distinct attachment rates influenced by WSS (125) or different biofilm coverage (128). Even within the same bacterial species, different strains can display discrepancies in attachment rates, particularly in the presence or absence of cellular motility (131). Additionally, when multiple bacterial species are involved, the impact of WSS can diverge (128). Current research also focuses on integrating novel features onto material surfaces, exploring their correlation with WSS and biofilm formation, such as brushes (122, 125, 127) and microstructures (120, 123). While certain approaches have demonstrated some benefits (123, 125), others have failed to show any significant improvement (120, 122). As of now, a flawless stent design has not yet been achieved, and none of the existing stents can completely prevent the formation of biofilms or encrustation on their surface.

## 5 Concluding remarks and outlook

Advanced materials and coatings: Continued exploration of novel materials and coatings could provide solutions to reduce encrustation and biofilm formation. Researchers might focus on materials that minimize friction during placement and removal while maximizing long-term performance.

Biofilm and encrustation control: Exploring innovative strategies, such as targeted antimicrobial/encrustation therapies or biofilm/encrustation-disrupting techniques, could offer new avenues to prevent stent-related complications.

Patient-specific and practice-driven design: For short-term stenting (less than two weeks), clinicians may prefer ureteral stents with better cost-effectiveness due to the small likelihood of encrustation and biofilm formation. For long-term stenting, a special focus on the current stent designs is crucial since small changes already proved beneficial in reducing attachment. Special care should be taken in the pigtail sections.

**Figure 2:**
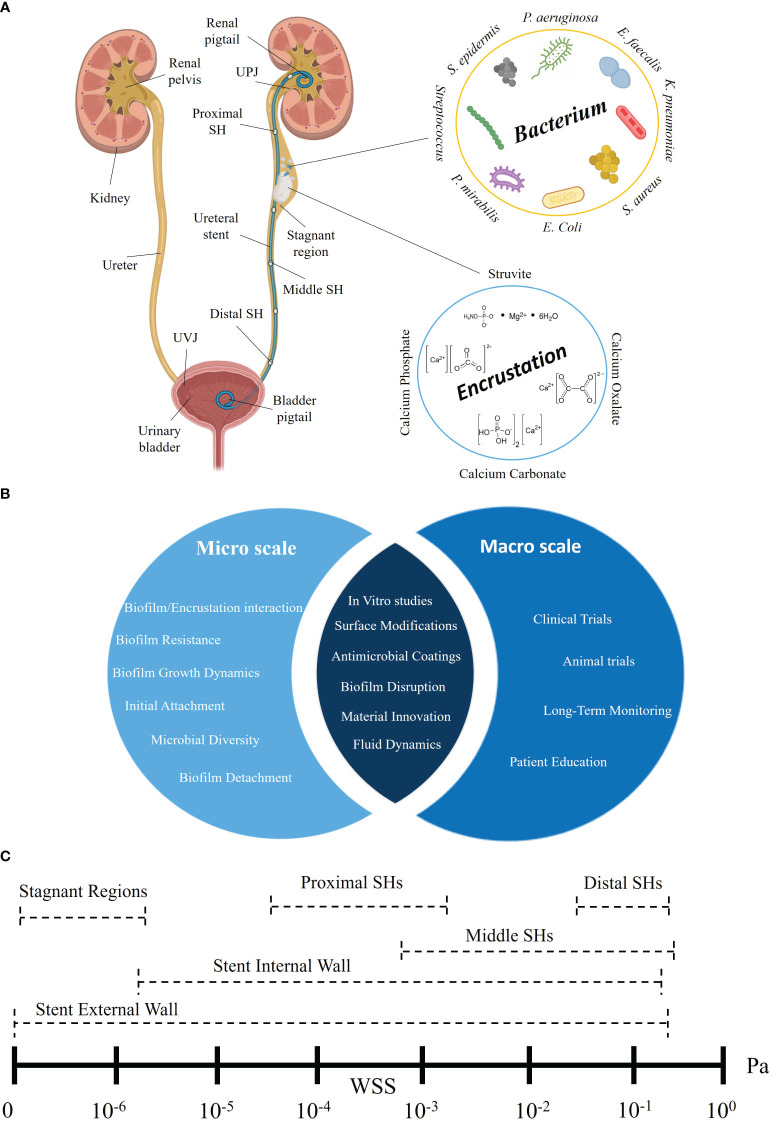
Multiscale analysis of biofilm formation and encrustation on ureteral stents: main bacteria, crystal, and wall shear stress distribution. **(A)** Schematic of the urinary tract with an obstructed stented ureter. Several sections of the ureteral stent, including the proximal side holes (near the UPJ), middle side holes (central part) and distal side holes (close to the UVJ), are represented. The figure highlights the main bacteria and crystals associated with indwelling ureteral stents. **(B)** Micro- and macro-scale investigations: research areas to tackle encrustation and biofilm formation across scales are represented. **(C)** WSS distribution: ranges of WSS at different ureteral stent locations, i.e., external and internal wall, stagnant regions, and proximal, middle, and distal side holes. *Illustrations in panel* **(A)** *were created with BioRender.com*.

Fluid dynamics and *in vitro* testing: Further advancements in mechanical modeling and *in vitro* flow analysis could provide deeper insights into urine flow patterns within the stented ureter. By simulating real-world conditions, researchers can better understand the impact of shear stress and flow patterns on stent performance. Research efforts could focus on developing methods to translate computational findings into real-world stent designs effectively. To this end, **Figure 2** illustrates an obstructed stented ureter and displays the main bacteria and crystals associated with ureteral stents. Micro- and macro-scale research areas are also presented, followed by a description of the WSS ranges within ureteral stents.

Innovative stents: New strategies and technologies are emerging, such as smart stents that incorporate sensors enabling real-time monitoring of urinary tract conditions. This could allow for early detection of complications and timely interventions.

Multidisciplinary approach: Encouraging collaboration between diverse disciplines, including engineering, microbiology, and urology, will be essential to tackle the challenges associated with stent complications.

Regulatory Guidelines: Collaboration between researchers and regulatory bodies could lead to the development of standardized guidelines for stent testing, evaluation, and reporting, promoting consistency in research.

## Author contributions

PA: Conceptualization, Writing – original draft, Methodology, Writing – review & editing. SZ: Conceptualization, Writing – review & editing, Methodology. DL: Writing – review & editing, Conceptualization. DC: Writing – review & editing, Conceptualization. SW: Writing – review & editing, Conceptualization. DO: Writing – review & editing, Conceptualization. FB: Writing – review & editing, Conceptualization. FC: Conceptualization, Funding acquisition, Writing – review & editing, Methodology, Supervision.
